# Pott’s disease as the initial presentation of tuberculosis: diagnostic challenges and radiological findings in district-level care—a case report

**DOI:** 10.1186/s13256-025-05728-8

**Published:** 2026-05-09

**Authors:** Thalente Mazibukwana

**Affiliations:** Oliver and Adelaide Tambo Regional Hospital, Bizana, Eastern Cape South Africa

**Keywords:** Spinal tuberculosis, Pott’s disease, Resource-limited, Empirical therapy, Diagnostic challenges, Kyphosis

## Abstract

**Background:**

Spinal tuberculosis (Pott’s disease) is a rare form of extra-pulmonary tuberculosis, often presenting with nonspecific symptoms and posing diagnostic challenges, especially in resource-limited settings. While microbiological confirmation is the gold standard, it is often unavailable in district hospitals.

**Case presentation:**

I report the case of a previously healthy 28-year-old Black female individual who presented with a 3-week history of progressive lower back pain, fatigue, and functional impairment but without pulmonary symptoms or known tuberculosis contacts. Examination revealed a lumbar spinal bulge, localized kyphosis, and tenderness. Laboratory studies showed elevated inflammatory markers (C-reactive protein, 157 mg/L; white blood cell count, 11.2 × 10^9^/L). A lateral lumbar spine X-ray demonstrated a wedge-shaped compression fracture at L3 with focal kyphotic deformity and endplate irregularity. Microbiological confirmation was not possible at the district level owing to a lack of advanced diagnostics. After multidisciplinary consultation, empirical antituberculous therapy was initiated per national guidelines. Subsequent computed tomography at a tertiary center strongly supported tuberculous spondylodiscitis, and the patient has shown marked clinical improvement on conservative management.

**Conclusion:**

This case illustrates the diagnostic complexity of spinal tuberculosis in district-level care and underscores the value of clinical suspicion, multidisciplinary collaboration, and careful assessment of plain radiographs when advanced diagnostics are unavailable. Clinicians should consider spinal tuberculosis in patients with chronic back pain and vertebral changes, even in the absence of classic tuberculosis symptoms. Early recognition and treatment are critical to prevent neurological compromise and deformity progression.

## Introduction

Tuberculosis (TB) remains a major global health challenge, with extrapulmonary forms accounting for a substantial proportion of cases, particularly in high-TB-burden regions [1]. Spinal tuberculosis (Pott’s disease) makes up 1–2% of all TB cases and up to 50% of musculoskeletal TB [2]. Delayed diagnosis is common owing to its insidious onset, nonspecific symptoms, and frequent lack of access to advanced imaging or microbiological confirmation in resource-limited settings [3]. At the district level, in low-resource settings, plain radiography is often the only available imaging modality. Although less sensitive than magnetic resonance imaging (MRI) and typically showing later changes (for example, anterior vertebral body destruction, disc-space narrowing, and progressive kyphosis), careful interpretation in the appropriate clinical context can strongly suggest tuberculous spondylodiscitis and guide timely initiation of therapy and referral for advanced imaging and surgical assessment when feasible. [4, 5]

I present a case of Pott’s disease in a young woman without pulmonary symptoms, highlighting the diagnostic challenges and decision-making based on clinical features and plain radiography at district-level care, with subsequent tertiary-level computed tomography (CT) support and conservative management.

## Case presentation

A 28-year-old Black woman with no significant past medical history presented to the district hospital with a 3-week history of progressive fatigue, lower back pain, inability to bend, and increasing reliance on a walking stick. She denied cough, shortness of breath, night sweats, fever, or weight loss, and there was no history of TB contact or immunosuppression. She experienced back pain and had normal spinal radiographs and received symptomatic treatment 1 year earlier. Human immunodeficiency virus (HIV) testing was negative. She developed a painless bulge on her lower back that gradually increased in size and became painful 10 months prior to this presentation.

### On examination

Vital signs: blood pressure 107/72 mmHg, pulse 133 beats per minute, temperature 36.5 °C, peripheral capillary oxygen saturation (SpO_2_) 98% (room air).

General: mild conjunctival pallor and no jaundice, cyanosis, clubbing, edema, or lymphadenopathy. Well-nourished and hydrated.

Neurological: no objective sensory or motor deficits; patient reported leg shaking after prolonged walking.

Musculoskeletal: visible and palpable bulge at L2–L4 with local tenderness and pain radiating to the thigh. Focal kyphosis was seen in the lumbar region.

Other systems: respiratory, cardiovascular, and abdominal examinations were unremarkable.

## Laboratory results

Sodium (Na): 135 mmol/L

Potassium (K): 4.1 mmol/L

C-reactive protein (CRP): 157 mg/L (elevated)

White blood cell (WBC) count: 11.2 × 10^9^/L (mildly raised)

Hemoglobin: 10.9 g/dL (mild anemia)

Platelets: 432 × 10^9^/L (high-normal/mild reactive thrombocytosis)

Urine analysis: proteinuria (2+)

## Radiological findings

### Lumbar spine X-ray in lateral view is shown in Fig. 1

**Fig. 1:**
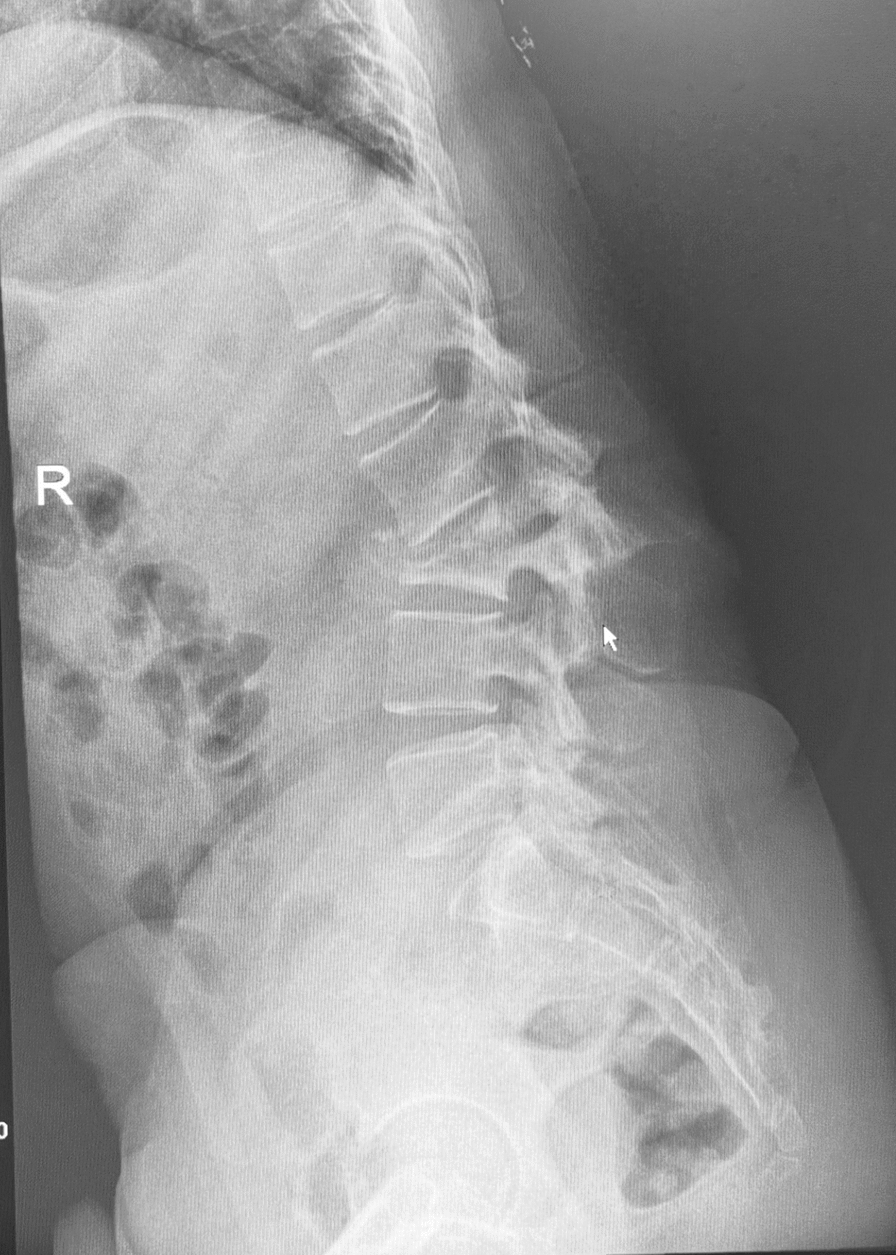
Lateral lumbar spine radiograph demonstrating a wedge-shaped compression fracture of L3 with focal kyphotic deformity and endplate irregularity. The loss of anterior vertebral height and endplate erosion are features compatible with tuberculous spondylodiscitis (Pott’s disease) in the appropriate clinical context. The white arrow indicates the collapsed L3 vertebral body and adjacent endplate changes

Lumbar spine radiograph showed a severe wedge-shaped compression fracture of the L3 vertebral body, with marked reduction in vertebral height, loss of the anterior cortex, collapse of the L3–L4 disc space, abnormal alignment with focal kyphosis, and irregularity of adjacent vertebral endplates (L2 and L4), suggesting contiguous involvement. No definite paraspinal soft-tissue shadow was appreciable on plain film. These radiologic findings are interpreted as consistent with tuberculous spondylodiscitis (Pott’s disease) in the clinical context. Image interpretation by the author is based on the attached original radiograph.

Figure 1 refers to the attached X-ray image and represents the author’s own interpretation.

### Diagnostic constraints

Advanced imaging (MRI/CT) and image-guided biopsy were unavailable locally. Microbiological confirmation was not feasible owing to the absence of pulmonary symptoms and lack of procedural capability.

## Differential diagnosis

- Neoplastic disease (primary bone tumor or metastasis)
- Pyogenic spondylodiscitis
- Osteoporotic fracture (unlikely owing to age and lack of risk factors)
- Spinal tuberculosis (favored by endemic area, chronicity, and radiological findings)

### Management

Following multidisciplinary discussion with a TB spine specialist at a tertiary center, a working diagnosis of spinal TB with focal kyphosis was made. Empirical antituberculous therapy was commenced according to national guidelines, with referral for advanced imaging and surgical evaluation.

## Outcome

At the 2-week follow-up, the patient reported improvement in pain and mobility. At the tertiary center, computed tomography strongly supported the diagnosis of tuberculous spondylodiscitis. The spine team recommended conservative management, noting early stage disease radiologically, and arranged a monthly follow-up at a specialized spine clinic.

By 4 months of antituberculous therapy, the patient reported significant improvement, including ambulation without assistive devices and substantial pain reduction. In collaboration with a TB specialist, the spine surgeon extended the planed duration of therapy to 12 months. Ongoing monitoring is focusing on neurological status, deformity progression, and treatment adherence.

Timeline:

**Table:** 

Time point	Event/findings	Action
T = −1 year	Episode of back pain, normal spinal X-ray	Symptomatic management
T = −10 months	Painless lumbar bulge, gradually enlarging	No imaging at that time
T = 0 (presentation at district)	Severe low back pain, focal kyphosis, CRP = 157 mg/L, WBC = 11.2 × 10^9^/L, platelets = 432 × 10^9^/L, urine analysis = 2+ proteinuria	Lateral lumbar X-ray obtained
T = +1 day	L3 wedge compression, narrowing disc space, kyphosis on X-ray	Multidisciplinary discussion, empirical anti-TB therapy initiated
T = +2 weeks	Pain and mobility improved	Referred to tertiary spine service
T = +1–2 months	CT at tertiary center strongly supporting TB of the spine	Conservative management, monthly spine clinic follow-ups
T = +4 months	Walking without aid, pain markedly reduced	Therapy continued, duration extended to 12 months
Planned to 12 months	Ongoing clinical/radiolographic monitoring	Continue conservative therapy, reassess for surgical indications if deterioration occurs

## Discussion

Spinal TB is the most common form of skeletal tuberculosis, predominantly affecting the thoracic and lumbar vertebrae [2, 4]. Its presentation is typically insidious, with localized back pain as the most frequent symptom. Constitutional features such as fever, night sweats, and weight loss may be absent, as in this case [5]. Neurological deficits are reported in up to 50% of cases but may be absent in early disease, resulting in diagnostic delays [6].

MRI is the preferred modality for early detection of vertebral involvement, epidural disease, and neural compromise; CT provides superior bony detail and assists biopsy planning [5–7]. In resource-limited settings, diagnosis relies heavily on clinical judgment, as advanced imaging (MRI and CT) and bacteriological confirmation are often unavailable [3]. Classical radiographic features are anterior vertebral body destruction, disc-space narrowing, and progressive kyphosis [7]. In this patient, the combination of chronic back pain, local swelling, focal kyphosis, elevated inflammatory markers, and wedge-compression fracture on X-ray was highly suggestive of Pott’s disease.

The novelty of this case lies in the necessity to make a diagnosis and initiate potentially life-saving therapy on the basis of clinical and basic radiological findings alone, in the absence of microbiological confirmation or advanced imaging—a scenario common in district-level hospitals in high-burden regions but underrepresented in medical literature. This case demonstrates the importance of maintaining a high index of suspicion and consulting with subspecialists, even in low-resource settings.

Empirical anti-TB therapy is reasonable in high-burden settings with compelling clinical and radiographic features, given the risk of irreversible neurological damage if treatment is delayed [8]. This approach carries risks, including overtreatment and missed alternative diagnoses; however, multidisciplinary input and early referral mitigate these. In this case, the chronicity, endemic context, and radiological findings supported spinal TB as the most likely diagnosis. Conservative measures (analgesia and/or physiotherapy) can support function, while surgical consultation is indicated for progressive deformity, instability, large abscesses, or neurological compromise.

Regarding ancillary laboratory findings, isolated 2+ proteinuria can accompany systemic inflammatory states; however, given potential renal implications in TB, repeat urine analysis and renal function monitoring are advisable. In our patient, there were no clinical features of nephrotic syndrome, and follow-up testing was recommended. The platelet count (432 × 10^9^/L) reflected a high-normal reactive thrombocytosis rather than thrombocytopenia, consistent with inflammatory activity.

## Limitations

- Microbiological confirmation and advanced imaging were unavailable.
- Empirical treatment carries a risk of mistreatment if the diagnosis is incorrect.
- Only early and intermediate follow-up are available to date, longer-term outcomes are being monitored at the tertiary center.

## Recommendations for practitioners

- In resource-limited, high-burden areas, clinicians should consider spinal TB in patients with chronic back pain and vertebral changes, even without classic pulmonary symptoms.
- Use plain radiography judiciously and evaluate for features suggestive of TB. Initiate empirical therapy when the index of suspicion is high and advanced diagnostics are unavailable.
- Arrange early referral to a multidisciplinary team and pursue definitive imaging/biopsy when feasible.
- Monitor closely for neurological deterioration, deformity progression, and treatment adherence.

## Learning points

- In resource-limited settings, plain radiography plus clinical findings may justify early empirical therapy for suspected spinal TB when advanced diagnostics are unavailable.
- Early recognition and coordinated referral are critical to avoid neurological complications and progressive kyphosis.
- Maintain diagnostic vigilance for alternative etiologies and secure microbiological and advanced imaging confirmation when feasible.

## Conclusion

This case illustrates the diagnostic complexity of spinal TB in resource-limited settings and underscores the value of clinical suspicion, multidisciplinary collaboration, and careful radiological assessment when advanced diagnostics are unavailable. Spinal TB should be suspected in patients with chronic back pain and vertebral abnormalities, particularly in TB-endemic regions, even when pulmonary symptoms are absent. Early recognition and treatment are critical to prevent neurological compromise and deformity progression.

## Data Availability

Not applicable.

## Abbreviations

TB: Tuberculosis
CRP: C-reactive protein
WBC: White blood cell
MRI: Magnetic resonance imaging
CT: Computed tomography
